# Systemic Approach to Identify Serum microRNAs as Potential Biomarkers for Acute Myocardial Infarction

**DOI:** 10.1155/2014/418628

**Published:** 2014-05-12

**Authors:** An Hsu, Shu-Jen Chen, Yu-Sun Chang, Hua-Chien Chen, Pao-Hsien Chu

**Affiliations:** ^1^Graduate Institute of Biomedical Sciences, School of Medicine, Chang Gung University, 259 Wen-Hwa 1st Road, Kwei-Shan, Tao-Yuan 333, Taiwan; ^2^Department of Biomedical Sciences, School of Medicine, Chang Gung University, 259 Wen-Hwa 1st Road, Kwei-Shan, Tao-Yuan 333, Taiwan; ^3^Molecular Medicine Research Center, Chang Gung University, 259 Wen-Hwa 1st Road, Kwei-Shan, Tao-Yuan 333, Taiwan; ^4^Department of Cardiology, Chang Gung Memorial Hospital, Chang Gung University College of Medicine, 199 Tun-Hwa North Road, Taipei 105, Taiwan; ^5^Healthcare Center, Chang Gung Memorial Hospital, Chang Gung University College of Medicine, 199 Tun-Hwa North Road, Taipei 105, Taiwan; ^6^Heart Failure Center, Chang Gung Memorial Hospital, Chang Gung University College of Medicine, 199 Tun-Hwa North Road, Taipei 105, Taiwan

## Abstract

*Background*. Recent studies have revealed the role of microRNAs (miRNAs) in a variety of biological and pathological processes, including acute myocardial infarction (AMI). We hypothesized that ST-segment elevation myocardial infarction (STEMI) may be associated with an alteration of miRNAs and that circulating miRNAs may be used as diagnostic markers for STEMI. *Methods*. Expression levels of 270 serum miRNAs were analyzed in 8 STEMI patients and 8 matched healthy controls to identify miRNAs differentially expressed in the sera of patients with AMI. The differentially expressed miRNAs were evaluated in a separate cohort of 62 subjects, including 31 STEMI patients and 31 normal controls. *Results*. The initial profiling study identified 12 upregulated and 13 downregulated serum miRNAs in the AMI samples. A subsequent validation study confirmed that serum miR-486-3p and miR-150-3p were upregulated while miR-126-3p, miR-26a-5p, and miR-191-5p were significantly downregulated in the sera of patients with AMI. Ratios between the level of upregulated and downregulated miRNAs were also significantly different in those with AMI. Receiver operator characteristics curve analysis using the expression ratio of miR-486-3p and miR-191-5p showed an area under the curve of 0.863. *Conclusion*. Our results suggest that serum miRNAs may be used as potential diagnostic biomarkers for STEMI.

## 1. Introduction

Cardiovascular disease is the leading cause of death for both men and women worldwide [1, 2]. According to the newly revised guidelines from the World Health Organization in 2000, a cardiac biomarker rise accompanied by typical symptoms or ST elevation is diagnostic of acute myocardial infarction (AMI) [3]. Percutaneous coronary intervention is one of the most important treatments for patients with ST-segment elevation myocardial infarction (STEMI) [2, 4, 5]. The door-to-balloon time should be less than 90 minutes, because any delay in time of reperfusion after arrival at the hospital is associated with a higher adjusted risk of in-hospital mortality [3, 5]. It would therefore be of great help to find biomarkers that could provide information on the pathophysiology and identify patients with STEMI to allow for early percutaneous coronary intervention.

In the first hours after AMI, myocardial fibers lose their transversal striations and nuclei. At the same time, there are robust upregulations of intramyocardial cytokines to enhance survival or accelerate myocyte necrosis and apoptosis and decrease contractility [6–8]. This is followed by cytokine amplification through transmigration of macrophages and neutrophils. The later cardiac remodeling includes phagocytosis and resorption of the necrotic tissue, hypertrophy of the surviving myocytes, degradation and synthesis of matrices, proliferation of myofibroblasts and angiogenesis, and, to a limited extent, progenitor cell proliferation [9, 10].

Recent studies have revealed the role of microRNAs (miRNAs) in a variety of basic biological and pathological processes [11–16] and in the association of miRNA signatures with cardiovascular diseases, including AMI [17–22]. Circulating miRNAs have been proposed to be sensitive and informative biomarkers for multiple cancers and in the diagnosis of cardiovascular diseases [23–28]. In particular, several muscle-specific miRNAs, including miR-1, miR-133a, and miR-133b, have been found to be significantly elevated in the sera of animals and patients with AMI. The identification of specific miRNAs acting as key regulators of AMI has opened new clinical avenues for research.

In this study, we first established a platform to quantify serum miRNAs, and we detected that the expression patterns of 25 miRNAs were altered in the sera of patients with AMI. Using an independent group, we confirmed that the serum levels of five miRNAs, including miR-486-3p, miR-191-5p, miR-126-3p, miR-26a-5p, and miR-150-3p, were significantly different in the AMI patients and that their expression levels can be used to differentiate AMI patients from normal patients. We further calculated the ratio between the upregulated and downregulated miRNAs and discovered that the ratio of these miRNAs had a much better predictive power to distinguish AMI patients from normal subjects. Taken together, our findings implicate circling miRNAs as potential diagnostic biomarkers for STEMI.

## 2. Methods

### 2.1. Patients' Data

This prospective study was conducted from November 2009 to January 2010 with approval from of the Institutional Regulation Board of Chang Gung Memorial Hospital, Taiwan, and it conformed to the tenets of the Declaration of Helsinki. In total, 39 consecutive patients with STEMI were enrolled. A presumptive diagnosis of STEMI was made based on the American College of Cardiology/American Heart Association Task Force on Practice Guidelines [1]. Thirty-nine age- and gender-matched normal controls who were undergoing routine medical examinations at the same hospital were also enrolled. All of the controls had normal physical and ocular examination results and no history of cardiovascular diseases [29]. The patient records were reviewed for demographic data and medical history [30, 31].  Table 1 lists the clinical characteristics of the healthy controls and AMI patients.

### 2.2. Sample Collection and RNA Preparation

Blood samples from the patients diagnosed with STEMI in the emergency department or the intensive care units were processed by two-step centrifugation. The supernatant was stored at −80°C. Total RNA was prepared from serum samples using TRIzol LS reagent (Invitrogen, Carlsbad, CA) according to the manufacture's protocol. In brief, 900 *μ*L of TRIzol LS reagent was added to 300 *μ*L of serum. The samples were mixed well and allowed to stand for 5 minutes at room temperature. A synthetic RNA (5′-CGAUGGGCAGCUAUAUUCACCUUG-3′) was added to the mixture as the spike-in control. After phase separation, the upper layer aqueous solution was transferred to a separate vial and RNA was precipitated with an equal volume of 2-propanol. The precipitation was carried out at −20°C for 1 hour to increase the yield of RNA. In general, we harvested approximately 300 ng of total RNA from each sample. The total RNA was then dissolved in 15 *μ*L of diethylpyrocarbonate-treated water, quantified by NanoDrop (Thermo Fisher Scientific Inc., Wilmington, USA), and stored at −80°C.

### 2.3. Reverse Transcription (RT)

A pulsed reverse transcription reaction was performed to convert all miRNAs into corresponding cDNAs in one RT reaction [32]. Briefly, 10 *μ*L of reaction mixture containing miRNA-specific stem-loop RT primers (final 2 nM each), 500 *μ*M dNTP, 0.5 *μ*L Superscript III (Invitrogen, Carlsbad, CA), and 3 *μ*L total RNA was used for the pulsed RT reaction, which was performed as follows: 16°C for 30 minutes, followed by 50 cycles at 20°C for 30 seconds, 42°C for 30 seconds, 50°C for 1 second, and 70°C for 10 minutes. The RT products were diluted 10-fold before being used for the miRNA quantitative real-time PCR reaction.

### 2.4. Quantitative Real-Time PCR (qPCR)

For miRNA quantification, 1 *μ*L of diluted RT product was used as the template for a 10 *μ*L qPCR. Briefly, 1X SYBR Master Mix (Applied Biosystems, Foster City, CA), 200 nM miRNA-specific forward primer, and 200 nM universal reverse primer were used for each qPCR reaction. The following conditions were used for qPCR: 95°C for 10 minutes, followed by 40 cycles of 95°C for 15 seconds and 63°C for 32 seconds, and a dissociation stage. End-point reaction products were analyzed on a 10% polyacrylamide gel stained with ethidium bromide to discriminate between the correct amplification product (57–60 bp) and the potential primer dimmers (<44 bp). All qPCR reactions were performed on an ABI Prism 7900 real-time PCR system (Applied Biosystems, Foster City, CA).

### 2.5. Data Analysis

The threshold cycle (Ct) for qPCR was defined as the cycle number at which the change of fluorescence intensity crossed the threshold of 0.2. Expression levels of miRNA were converted to 39-Ct [32]. For the profiling study, the expression data were normalized by global median normalization before further analysis. For the validation studies, the synthetic spiked-in miRNA was used for normalization.

### 2.6. Statistical Analysis

Quantitative data were expressed as mean ± standard deviation and analyzed using the Student's *t*-test. Receiver operator characteristics (ROC) curves and areas under the curves (AUC) were calculated using Prism 5 software (GraphPad). Prism calculates *z* = (AUC − 0.5)/SE area and then determines *P* values from the *z* ratio (normal distribution). Cutoff values corresponded to the highest sum of sensitivity and specificity. A *P* value less than 0.05 was considered to be statistically significant. The statistical analyses used for miRNA expression data, including the *t*-test (two-tailed), principle component analysis, and hierarchical clustering, were performed with the Partek Genomics Suite (version 6.3, St. Louis, MO).

## 3. Results

### 3.1. Identification of Differentially Expressed miRNAs in the Sera from the STEMI Patients and Healthy Controls

**Figure 1:**
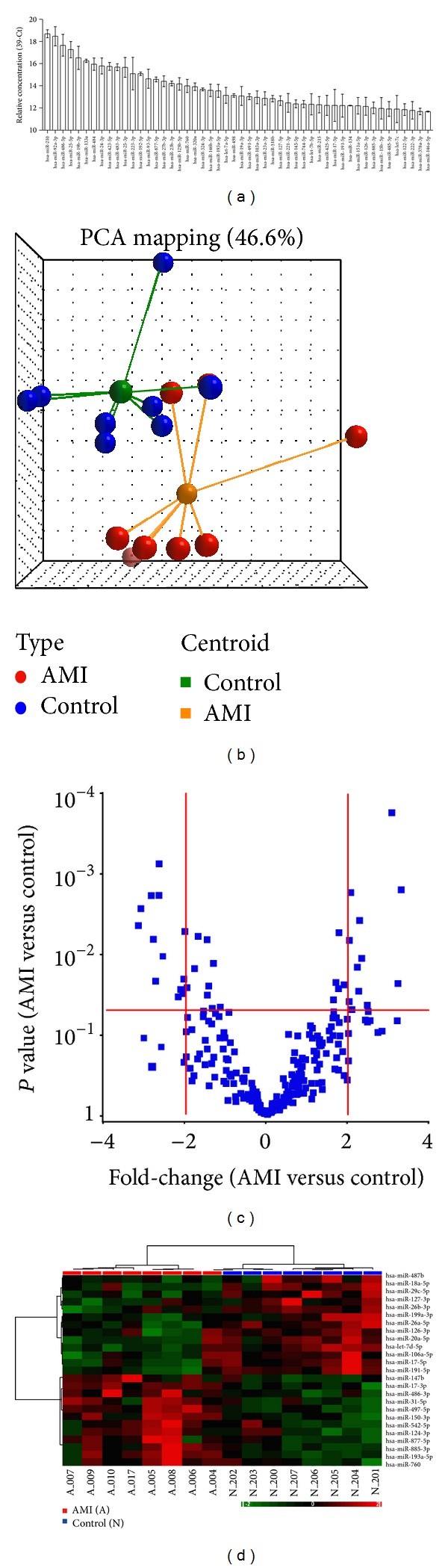
Detection of differentially expressed circulating miRNAs in serum samples. (a) Detection of miRNAs in serum from healthy subjects. Expression levels of 270 miRNAs in 300 *μ*L of serum were quantified using a multiplexed RT-qPCR assay. The relative expression levels of the top 50 miRNAs detected in serum from three healthy subjects (mean ± SD) are shown. (b) Principle component analysis using expression levels of the 270 human miRNAs in serum samples from 8 healthy (blue) and 8 AMI (red) subjects. (c) Volcano plot indicated that 25 miRNAs were significantly altered in sera from the AMI patients. Red lines indicate the Student's *t*-test *P* = 0.05 and ±2-fold change. (d) Unsupervised hierarchical clustering of healthy and AMI samples using the 25 differentially expressed serum miRNAs. The hierarchical clustering was generated using Pearson's dissimilarity as the distance measure and Ward's method for linkage analysis. A: AMI patients; N: healthy subjects.

Recent studies have identified several cardiac-specific or -enriched miRNAs in the circulation as potential biomarkers for the diagnosis of AMI. As AMI is a complex disease, it is possible that additional circulating miRNAs may also be altered and can then be used as potential markers for AMI. To explore this possibility, we quantified the expression levels of 270 miRNAs in serum samples from 8 healthy controls and 8 patients with STEMI using a qPCR assay platform previously established in our laboratory [32]. The clinical characteristics of the subjects in the validation study are shown in Table 1. In order to detect a large number of miRNAs from small quantities of serum, we implemented a multiplexed reverse transcription reaction and used SYBR Green-based qPCR method for miRNA quantification. The assay was modified from the stem-loop RT-PCR assay originally designed by Chen et al. [33]. The SYBR Green-based RT-qPCR assay for miRNA detection has been reported by other laboratories [34]. A pilot study using RNA prepared from 300 *μ*L of serum samples from three healthy subjects detected approximately 100 miRNAs with high confidence (Ct < 32) in each sample, similar to the results reported by Wang et al. using the TaqMan method [23]. The expression levels of the serum miRNAs detected in our assay also correlated well with the expression levels of serum miRNAs reported by Wang et al. [23] (Figure 1(a)), suggesting that the SYBR Green-based RT-qPCR assay was suitable for the profiling study.

Expression levels of the 270 miRNAs were normalized and used to identify differentially expressed miRNAs. As there is currently no consensus on the best internal control for circulating miRNA profiling analysis, global median normalization was used to correct the technical variations arising from RNA preparation and qPCR detection. Principle component analysis revealed that the overall expression pattern of the 270 miRNAs in the sera from the patients with AMI was significantly different from the healthy controls (Figure 1(b)). Using the criteria of *P* < 0.05 (*t*-test, two-tailed) and fold-change ≥2, we identified 25 serum miRNAs whose expression levels were significantly altered in the AMI samples (Figure 1(c)). Unsupervised hierarchical clustering using the expression levels of these 25 serum miRNAs completely separated the AMI patients from the healthy controls (Figure 1(d)).

The list of differentially expressed serum miRNAs, including 12 upregulated and 13 downregulated miRNAs, is shown in Table 2. These results confirmed that sera from the AMI patients contained multiple differentially expressed miRNAs and suggested that some of these differentially expressed miRNAs could be used as markers for the diagnosis of AMI. Previous studies have identified several serum miRNAs, including miR-1, miR-133, and miR-208, whose levels are significantly increased in the sera from AMI patients and experimental animals [23, 24, 35]. In our profiling study, levels of both miR-1 and miR-208 were found to be increased in sera from the AMI patients. miR-1 showed a 1.46-fold increase (*P* = 0.206) and miR-208 showed a 1.87-fold increase (*P* = 0.074) in the AMI samples. In contrast, we did not detect any difference in the level of miR-133 between the AMI and control subjects (fold-change = −1.042, *P* = 0.819). This result is consistent with the study by Ai et al. [24].

### 3.2. Validation of Candidate miRNAs in an Independent Cohort

**Figure 2:**
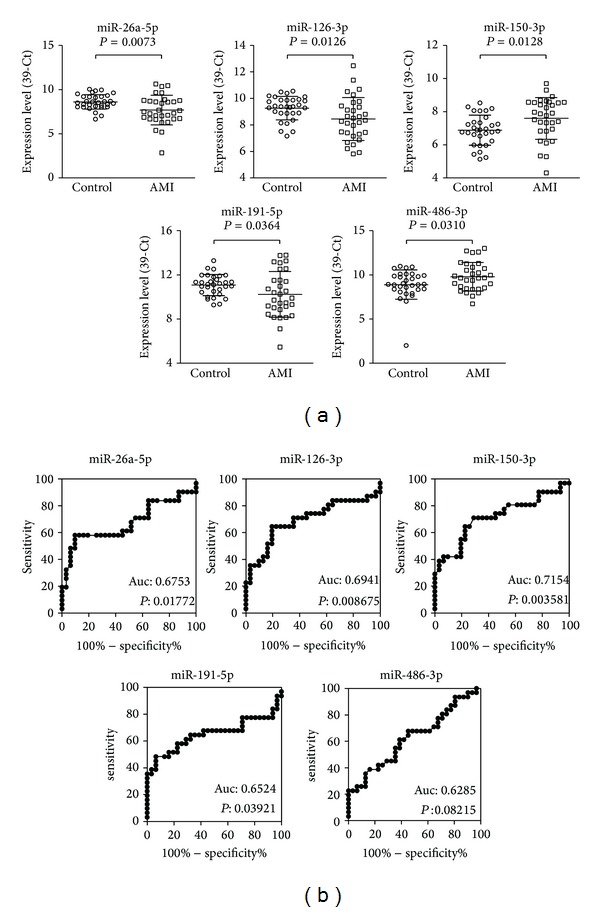
Expression levels and predictive power of five candidate miRNAs in the healthy controls and acute myocardial infarction (AMI) patients. (a) Expression levels of 5 candidate miRNAs in serum samples from 31 healthy controls and 31 AMI patients. Ct values generated from RT-qPCR were normalized to the spiked-in synthetic miRNA and then converted to 39-Ct. Data are presented as mean ± SD. *P* values were calculated using the *t*-test. (b) ROC analysis using expression levels of individual miRNAs in healthy controls and AMI samples.

To determine whether the differentially expressed serum miRNAs could be used as blood-based biomarkers to differentiate AMI patients from healthy subjects, we conducted a validation study of the 25 differentially expressed miRNAs using an independent cohort including 31 AMI patients and 31 age- and gender-matched healthy controls. The expression levels of these candidate miRNAs were quantified using multiplexed RT-qPCR, and a synthetic miRNA was used as a spike-in control to correct the variations in sample preparation and RT-qPCR. The expression data of the miRNAs were normalized to the spike-in control before further analysis. Five of the candidate miRNAs (2 upregulated and 3 downregulated) showed statistically significantly different expressions (*t*-test, *P* < 0.05, fold-change > 1.5) in the AMI samples compared to the healthy controls.  Figure 2(a) shows the expression levels of these five validated miRNAs in individual samples from AMI patients and healthy controls.

To evaluate the diagnostic value of these validated miRNAs, ROC curves were constructed and the AUC values were determined (Table 3, Figure 2(b)). Among the 5 validated miRNAs, miR-150-3p, which was upregulated in the AMI samples, showed the best diagnostic power with an AUC of 0.715 ± 0.067 (*P* = 0.0036) and a 95% confidence interval of 0.584 to 0.847. The cutoff value for miR-150-3p was 7.29 with a sensitivity of 70.97% and a specificity of 70.97%. miRNA miR-126-3p, which was downregulated in the AMI samples, showed the second best diagnostic power with an AUC of 0.694 ± 0.070 (*P* = 0.0087) and a 95% confidence interval of 0.557 to 0.832. The cutoff value for miR-126-3p was 8.835 with a sensitivity of 60.52% and a specificity of 80.65%. Interestingly, previous studies have shown that miR-150 is an inflammatory-related miRNA while miR-126 is associated with angiogenesis. These results suggest that miRNAs associated with pathological conditions other than cardiac damage may also be useful as biomarkers for the diagnosis of AMI.

### 3.3. Evaluation of miRNA Expression Ratio as a Biomarker for AMI

**Figure 3:**
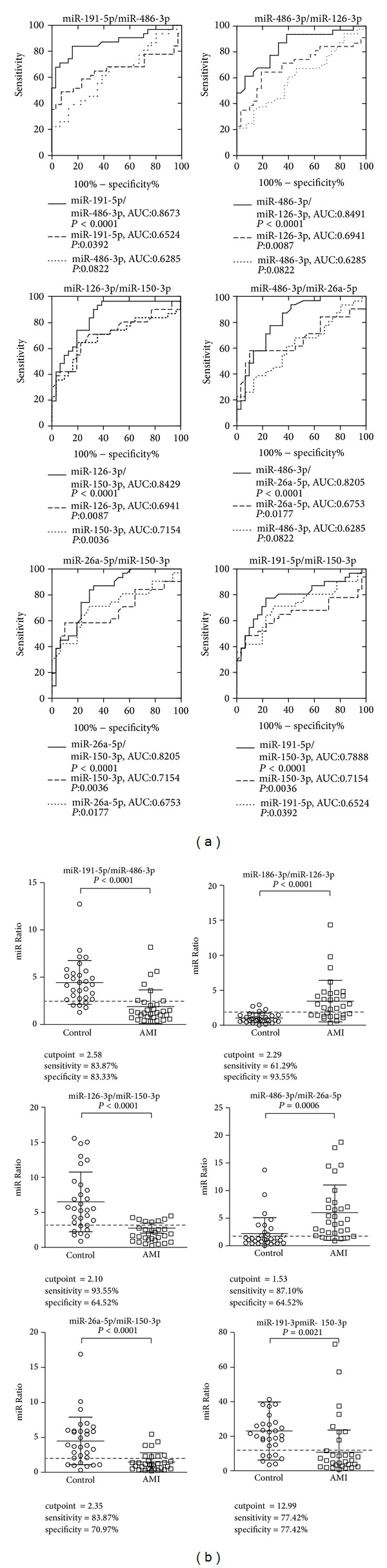
Predictive power and expression ratios between two candidate serum miRNAs in the healthy controls and acute myocardial infarction (AMI) patients. (a) ROC analysis using expression ratios between two miRNAs (control *N* = 31, AMI *N* = 31 patients). (b) Expression ratios of miRNA combinations in AMI and control samples. Data are presented as mean ± SD. *P* values were calculated using the *t*-test.

Previous studies have shown that the expression ratio of two miRNAs provides a better predictive power in the diagnosis of head and neck cancer [36]. As miR-150-3p and miR-486-3p showed significant upregulation and miR-26a-5p, miR-126-3p, and miR-191-5p showed significant downregulation in the AMI samples, we next sought to determine if expression ratios constructed between these miRNAs could improve their predictive power for the diagnosis of AMI. For each miRNA pair, the expression ratio was determined by calculating the ΔCt values between the upregulated and downregulated miRNAs. The expression ratios of six different combinations between the 2 upregulated and 3 downregulated miRNAs were then evaluated for their diagnostic accuracy using ROC analysis (Figure 3(a)).

Remarkably, all six different expression ratios had much better predictive power than individual miRNAs with AUC *P* values less than 0.0001 (Table 4). Five of the six expression ratios showed an AUC greater than 0.8. The expression ratio between miR-486-3p and miR-191-5p showed the best predictive power, with an AUC of 0.863 and 95% confidence interval of 0.765 to 0.961. The optimal cutoff value was 2.58 with a sensitivity of 83.87% and a specificity of 83.33% (Figure 3(b)). Although the sample size was small, this study provides strong support for the notion that circulating miRNAs may be used as diagnostic markers. More importantly, these data clearly demonstrated the superior differentiating power of miRNA ratios rather than single miRNA levels as biomarkers for the diagnosis of AMI.

## 4. Discussion 

miRNAs represent an abundant group of small noncoding RNAs that regulate gene expression and affect physiological processes such as development, cell proliferation, and cell death [37, 38]. miRNAs may respond to damage in cardiovascular diseases, such as in acute responses to ischemia [17, 19–21], in chronic stages such as hypertrophy [39–44], and in heart failure [37, 44–48]. Studies of miRNAs in cardiovascular diseases have mostly been based on tissues of disease models [41, 44, 45, 49] and transgenic mice [39, 40, 44, 46]. The possibility of using miRNAs as a novel myocardial biomarker has been previously raised [50]; however, studies on the detection of blood miRNAs in AMI patients are limited [23]. One study showed that miR-208 was significantly increased after isoproterenol-induced myocardial injury [35], and another study demonstrated that circulating miR-1 was a potential novel biomarker for AMI [24].

In our profiling study, we confirmed the increased expression of miR-1 and miR-208 in AMI serum. In addition, we identified several circulating miRNAs whose expression was even more profoundly altered in AMI patients. Two of the most significantly upregulated miRNAs observed in the AMI samples were miR-486-3p and miR-150-3p. Similar to miR-1 and miR-133, miR-486 is a muscle-enriched miRNA [51]. Recent studies have found that miR-486 is controlled by three transcription factors known to regulate muscle growth and homeostasis, including SRF, MRTF-A, and MyoD [51]. The observation that miR-486 is downregulated in Duchenne's muscular dystrophy [52] and in denervation-induced muscle atrophy [51] further supports that miR-486 is a critical regulator for muscle growth. Small et al. showed that miR-486 directly targets phosphatase and tensin homolog (PTEN) and Foxo1a to enhance the PI3 K/AKT signaling in muscle cells [51]. Overexpression of miR-486 reduces the protein level of PTEN and Foxo1a and enhances PI3 K/AKT signaling, eventually leading to muscle hypertrophy [51]. PI3 kinase is a known regulator of skeletal muscle hypertrophy and atrophy [53]. As left ventricular hypertrophy often precedes AMI [54], the increase of miR-486 in the plasma of AMI patients may reflect an underlying cardiac hypertrophy associated with these AMI patients. Recent studies have shown that pharmacologic inhibition of PI3 K gamma promotes infarct resorption and prevents adverse cardiac remodeling after myocardial infarction in mice [55]. It is possible that pharmacologic inhibitors blocking the PI3 K/AKT signaling may also provide benefit for AMI patients with increased miR-486 level.

miR-150 is highly expressed in immune cells, including B- and T-lymphocytes, and has been shown to regulate the proliferation and differentiation of myeloid and lymphoid cells [56]. Recently, dysregulated expression of miR-150 has been reported in cardiac tissues from AMI patients [25, 57], consistent with our observation that plasma miR-150-3p levels were upregulated in the AMI samples. miR-150 has been shown to aggravate H_2_O_2_-induced cardiac myocyte injury by downregulating c-Myb gene [58], a gene involved in regulating the differentiation of myogenic progenitor cells. These results suggest that miR-150 may participate in H_2_O_2_-mediated gene regulation and functional modulation in cardiac myocytes.

The profiling analysis revealed that a large number of miRNAs are downmodulated in AMI, including miR-126 and miR-26. The most significantly downregulated miRNA was the endothelial cell-specific miR-126, which promotes angiogenesis in response to angiogenic growth factors, such as vascular endothelial growth factor or basic fibroblast growth factor, by repressing negative regulators of signal transduction pathways and inflammation [45, 59–63]. Previously, Jakob et al. reported a pronounced loss of miR-126 in angiogenic early outgrowth cells (EOCs, CD34^+^) in patients with chronic heart failure [45]. The authors observed that miR-126 mimic transfection increased the capacity of angiogenic EOCs from patients with CHF to improve cardiac neovascularization and function. Recently, Qiang et al. also reported that dysregulated miR-126 in endothelial progenitor cells is associated with the prognosis of chronic heart failure patients [64]. These results suggest that administration of miR-126 may rescue endothelial cell function and offer a potential therapeutic approach.

In this study, we observed a significantly reduced miR-26 level in AMI samples. Zhang et al. recently found that miR-26 is significantly reduced in a rat cardiac hypertrophy model and may regulate physiological structural changes of rat hearts by targeting glycogen synthase kinase 3 *β* (GSK3*β*) [65]. The authors suggested that overexpression of miR-26 or suppression of GSK3*β* may represent a promising therapeutic strategy. Although no previous study has linked miR-191 to cardiovascular functions, the expression ratio between miR-191 and miR-486 may serve as a potential serum biomarker for AMI patients in our study.

The major limitation of the current study is that we could not record the exact onset time of STEMI in our patients. A difference in AMI onset could contribute to the variation of serum miRNA levels among these patients. However, our data are mostly compatible with the previous AMI studies [1, 6, 19, 23, 24].

## 5. Conclusion

In conclusion, we found that the serum level of five miRNAs, including miR-486-3p, miR-191-5p, miR-126-3p, miR-26a-5p, and miR-150-3p, were significantly different in the AMI patients compared to the healthy controls. ROC analysis using the expression ratio of miR-486-3p and miR-191-5p showed an area under the serum concentration time curve of 0.867. Our findings implicate that serum miRNAs may be used as potential diagnostic biomarkers for STEMI.

## Figures and Tables

**Table 1:** Characteristics of the patients.

	Exploration cohort			Validation cohort		
	Ctrl (*n* = 8)	AMI (*n* = 8)	*P* value	Ctrl (*n* = 31)	AMI (*n* = 31)	*P* value
Age (years)	44.6 ± 10.3	53.3 ± 9.2	0.0980	53.7 ± 14.8	59.0 ± 11.5	0.1235
Male/female (*n*/*n*)	6/2	8/0		29/2	29/2	
Hypertension (%)	16	20	0.2873	17	19	0.323
Diabetes mellitus	12	14	0.765	11	14	0.635
Hypercholesterolemia	12	14	0.864	14	16	0.123
Smoking	14	14	0.125	14	15	0.110
Troponin I (<0.2 ng/mL)	NA	25.9 ± 65.3		NA	11.7 ± 28.2	
Triglyceride (mg/dL)	138.9 ± 73.7	144.0 ± 52.9	0.8750	161.1 ± 94.1	154.0 ± 86.7	0.7685
Cholesterol (mg/dL)	187.0 ± 26.1	164.6 ± 30.9	0.1400	197.9 ± 41.2	160.9 ± 37.8	0.0008
White blood cells (×10^3^/uL)	6.35 ± 1.76	11.19 ± 2.19	<0.0001	6.48 ± 1.84	11.15 ± 3.99	<0.0001
Creatinine (md/dL)	0.91 ± 0.22	0.95 ± 0.13	0.6560	0.96 ± 0.22	1.28 ± 1.20	0.1505

Ctrl: control; AMI: acute myocardial infarction; NA: not available; Troponin I was the highest value.

**Table 2:** miRNAs differentially expressed in the sera from patients with acute myocardial infarction.

miRNA name	Chromosome location	AMI (mean ± SD)	Ctrl. (mean ± SD)	Fold-change (AMI versus Ctrl.)	*t*-test *P* value
miRNA upregulated in AMI					
miR-193a-5p	17q12	13.15 ± 1.02	11.49 ± 0.48	3.17	0.0016
miR-147b	15q21.1	5.96 ± 1.47	4.34 ± 0.85	3.07	0.0232
miR-497-5p	17p13.1	9.12 ± 0.57	7.58 ± 0.57	2.91	0.0002
miR-542-5p	Xq26.3	6.34 ± 1.07	5.09 ± 1.05	2.36	0.0431
miR-885-3p	3p25.3	11.05 ± 0.99	9.88 ± 0.42	2.25	0.0113
miR-150-3p	19q13.32	9.87 ± 0.66	8.72 ± 0.58	2.21	0.0038
miR-877-5p	6p21.33	13.40 ± 1.06	12.26 ± 0.66	2.20	0.0290
miR-31-5p	9p21.3	7.74 ± 0.84	6.63 ± 0.66	2.17	0.0145
miR-760	1p22.1	13.01 ± 0.91	11.96 ± 0.94	2.07	0.0490
miR-17-3p	13q31.3	7.24 ± 0.23	6.21 ± 0.67	2.04	0.0017
miR-486-3p	8p11.21	9.19 ± 0.66	8.17 ± 0.54	2.02	0.0068
miR-124-3p	8p23.1, 8q12.3	7.23 ± 0.79	6.21 ± 0.90	2.02	0.0390

miRNA downregulated in AMI					
miR-20a-5p	13q31.3	7.20 ± 1.00	8.79 ± 0.76	−3.01	0.0045
miR-18a-5p	13q31.3	4.86 ± 0.83	6.41 ± 0.78	−2.94	0.0027
miR-26a-5p	12q14.1, 3p22.2	7.96 ± 0.75	9.38 ± 0.65	−2.69	0.0019
miR-17-5p	13q31.3	8.36 ± 0.92	9.76 ± 0.72	−2.64	0.0066
miR-106a-5p	Xq26.2	6.82 ± 1.15	8.19 ± 0.84	−2.59	0.0216
let-7d-5p	9q22.32	8.44 ± 0.66	9.77 ± 0.65	−2.52	0.0019
miR-191-5p	3p21.31	9.68 ± 0.62	11.01 ± 0.54	−2.51	0.0008
miR-26b-3p	2q35	6.62 ± 1.09	7.91 ± 0.42	−2.44	0.0107
miR-126-3p	9q34.3	9.15 ± 1.17	10.25 ± 0.44	−2.14	0.0340
miR-487b	14q32.31	5.10 ± 0.56	6.16 ± 1.01	−2.09	0.0278
miR-127-3p	14q32.31	6.25 ± 0.71	7.29 ± 0.93	−2.06	0.0313
miR-199a-3p	1q25.1, 19p13.2	7.31 ± 0.60	8.34 ± 0.86	−2.04	0.0205
miR-29c-5p	1q32.2	7.56 ± 0.56	8.57 ± 0.60	−2.02	0.0052

Ctrl.: control; and AMI: acute myocardial infarction.

**Table 3:** Validation of differentially expressed serum miRNAs in patients with acute myocardial infarction.

miRNA name	AMI (mean ± SD)	Ctrl. (mean ± SD)	Fold-change (AMI versus Ctrl.)	*t*-test *P* value	ROC AUC	ROC *P* value
miRNA upregulated in AMI						
miR-486-3p	9.79 ± 1.61	8.89 ± 1.66	1.87	0.0310	0.6285	0.0822
miR-150-3p	7.60 ± 1.25	6.88 ± 0.91	1.64	0.0128	0.7154	0.0036

miRNA downregulated in AMI						
miR-26a-5p	7.68 ± 1.68	8.61 ± 0.84	−1.90	0.0073	0.6753	0.0177
miR-191-5p	10.23 ± 2.07	11.08 ± 0.96	−1.80	0.0364	0.6524	0.0392
miR-126-3p	8.45 ± 1.61	9.27 ± 0.89	−1.76	0.0126	0.6941	0.0087

Ctrl.: control; AMI: acute myocardial infarction; ROC: receiver operator characteristics; and AUC: area under the curve.

**Table 4:** Receiver operator characteristic curve analysis of miRNA ratios in the prediction of acute myocardial infarction.

Combined miRNAs	AMI (mean ± SD)	Ctrl. (mean ± SD)	*t*-test *P* value	ROC AUC	AUC 95% CI	ROC *P* value	Sensitivity	Specificity
miR-191-5p/miR-486-3p	1.89 ± 1.78	4.42 ± 2.31	1.02*E* − 05	0.8629	0.7648 to 0.9610	<0.0001	83.87%	83.33%
miR-486-3p/miR-126-3p	3.46 ± 2.96	1.06 ± 0.71	4.66*E* − 05	0.8491	0.7531 to 0.9451	<0.0001	61.29%	93.55%
miR-126-3p/miR-150-3p	2.77 ± 4.04	6.52 ± 4.24	7.0*E* − 04	0.8429	0.7423 to 0.9434	<0.0001	93.59%	64.52%
miR-486-3p/miR-26a-5p	6.00 ± 5.03	2.23 ± 2.88	0.0006	0.8205	0.7160 to 0.9250	<0.0001	77.42%	74.19%
miR-26a-5p/miR-150-3p	1.50 ± 1.34	4.50 ± 3.41	2.49*E* − 05	0.8205	0.7166 to 0.9244	<0.0001	83.87%	70.97%
miR-191-5p/miR-150-3p	10.77 ± 12.84	23.01 ± 16.87	0.0021	0.7888	0.6718 to 0.9058	<0.0001	77.42%	77.42%

Ctrl.: control; AMI: acute myocardial infarction; ROC: receiver operator characteristics; and AUC: area under the curve.
